# Early operative management in patients with adhesive small bowel obstruction: population‐based cost analysis

**DOI:** 10.1002/bjs5.50311

**Published:** 2020-06-30

**Authors:** R. Behman, A. B. Nathens, P. Pechlivanoglou, P. Karanicolas, J. Jung, N. Look Hong

**Affiliations:** ^1^ Division of General Surgery, Department of Surgery Toronto Ontario Canada; ^2^ Institute of Health Policy Management and Evaluation University of Toronto Toronto Ontario Canada; ^3^ Division of General Surgery, Sunnybrook Health Sciences Centre Toronto Ontario Canada; ^4^ Child Health Evaluative Sciences, The Hospital for Sick Children Toronto Ontario Canada

## Abstract

**Background:**

Adhesive small bowel obstruction (aSBO) is a potentially recurrent disease. Although non‐operative management is often successful, it is associated with greater risk of recurrence than operative intervention, and may have greater downstream morbidity and costs. This study aimed to compare the current standard of care, trial of non‐operative management (TNOM), and early operative management (EOM) for aSBO.

**Methods:**

Patients admitted to hospital between 2005 and 2014 in Ontario, Canada, with their first episode of aSBO were identified and propensity‐matched on their likelihood to receive EOM for a cost–utility analysis using population‐based administrative data. Patients were followed for 5 years to determine survival, recurrences, adverse events and inpatient costs to the healthcare system. Utility scores were attributed to aSBO‐related events. Cost–utility was presented as the incremental cost‐effectiveness ratio (ICER), expressed as Canadian dollars per quality‐adjusted life‐year (QALY).

**Results:**

Some 25 150 patients were admitted for aSBO and 3174 (12·6 per cent) were managed by EOM. Patients managed by TNOM were more likely to experience recurrence of aSBO (20·9 per cent *versus* 13·2 per cent for EOM; *P* < 0·001). The lower recurrence rate associated with EOM contributed to an overall net effectiveness in terms of QALYs. The mean accumulated costs for patients managed with EOM exceeded those of TNOM ($17 951 *versus* $11 594 (€12 288 *versus* €7936) respectively; *P* < 0·001), but the ICER for EOM *versus* TNOM was $29 881 (€20 454) per QALY, suggesting cost‐effectiveness.

**Conclusion:**

This retrospective study, based on administrative data, documented that EOM may be a cost‐effective approach for patients with aSBO in terms of QALYs. Future guidelines on the management of aSBO may also consider the long‐term outcomes and costs.

## Graphical abstract

This population‐based cost–utility analysis compared early operative management (EOM) for adhesive small bowel obstruction (SBO) to the current standard of care of a trial of non‐operative management. Although EOM was more costly, it was associated with a significantly lower risk of recurrence and reduced exposure to the morbidity and costs associated with multiple admissions for adhesive SBO. With longer follow‐up, EOM becomes increasingly cost‐effective, and crosses published willingness‐to‐pay thresholds within 5 years of the first admission.

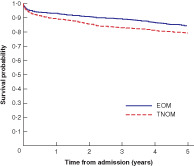

Early operative management in small bowel occlusion

## Abstract (es)

**Antecedentes:**

La oclusión de intestino delgado por adherencias (*adhesive small bowel obstruction*, aSBO) es una enfermedad potencialmente recidivante. Aunque el tratamiento no quirúrgico es a menudo eficaz, se asocia con un mayor riesgo de recidiva que la intervención quirúrgica, y puede provocar más adelante morbilidad y costes. El objetivo de este estudio fue comparar un Ensayo de Tratamiento No Quirúrgico (*Trial of Non‐operative Management*, TNOM, el estándar actual de tratamiento) con Tratamiento Operatorio Precoz (*Early Operative Management*, EOM) para el tratamiento de aSBO.

**Métodos:**

Pacientes ingresados en el hospital entre 2005‐2014 en Ontario, Canadá con un primer episodio de aSBO fueron identificados y emparejados por puntaje de propensión respecto a la probabilidad de recibir EOM para un análisis de coste‐utilidad utilizando datos administrativos de base poblacional. Los pacientes fueron seguidos durante 5 años para determinar la supervivencia, recidivas, eventos adversos, y costes de la hospitalización para el sistema de salud. Las puntuaciones de utilidad se atribuyeron a los eventos relacionados con la aSBO. El coste‐utilidad se presentó como la razón costo efectividad incremental (*incremental cost‐effectiveness ratio*, ICER) expresada como dólares por año de vida ajustado por calidad (*quality‐adjusted life‐year*, QALY).

**Resultados:**

Un total de 25.150 pacientes fueron ingresados por aSBO y 3.174 (12,6%) fueron tratados con EOM. Los pacientes tratados mediante TNOM tenían más probabilidades de presentar una recidiva de la aSBO (20,9% *versus* 13,2%, *P* < 0,0001). La menor incidencia de recidivas asociada con EOM contribuyó a una eficacia neta global en términos de QALYs. Mientras que los costes medios acumulados para los pacientes tratados con EOM superaron a los de TNOM ($17,951 *versus* $11,594, *P* < 0,0001), el ICER de EOM *versus* TNOM fue $29,881/QALY, lo que sugiere un coste‐eficacia de esta estrategia.

**Conclusión:**

Este estudio retrospectivo basado en datos administrativos evidenció que EOM puede representar un abordaje coste‐efectivo para pacientes con aSBO en términos de QALYs. Las futuras guías clínicas para el tratamiento de la aSBO pueden también considerar los resultados a largo plazo y los costes.

## Introduction

Small bowel obstruction (SBO) is among the most common reasons for admission to a surgical service in developed countries, accounting for 12–16 per cent of surgical admissions^1^. Approximately 75 per cent of SBOs are caused by intra‐abdominal adhesions^2^. The burden on healthcare systems associated with adhesive SBO (aSBO) is considerable; in the USA, more than 350 000 operations for aSBO are performed annually, resulting in more than US $2·3 billion (€2·1 billion; exchange rate 19 May 2020) in healthcare expenditure^3^.

Current guidelines advocate a trial of non‐operative management (TNOM) for aSBO in patients with no signs of bowel ischaemia or sepsis. Suggested trials of non‐operative management typically range from 3 to 5 days, after which surgical intervention is undertaken^4^. Approximately 70–80 per cent of patients with aSBO experience resolution of their symptoms without surgical intervention^4^.

Although non‐operative management is often successful with respect to the acute admission, there is a near twofold increase in the risk of recurrence of aSBO after non‐operative management, compared with operative management^5^, ^6^. With each recurrence, the probability of an additional subsequent recurrence increases and the time between recurrences shortens^5^. Each admission for aSBO carries considerable costs and risks, with an average length of stay of 8 days and short‐term mortality rate of 5–10 per cent^7^, ^8^, ^9^. Recent evidence^10^ has suggested that non‐operative management of aSBO may be associated with a significantly poorer long‐term survival, as the increased incidence of recurrence results in greater exposure to the risks associated with hospitalization for aSBO. Moreover, for patients in whom a trial of non‐operative management fails, the delay in surgery is associated with an increased risk of morbidity and mortality^11^, ^12^.

Early operative management (EOM) for aSBO may reduce the risks associated with delayed operation and reduce the risk of recurrence. Although EOM may be associated with greater upfront costs and morbidity associated with operative intervention, these costs and risks might be outweighed by fewer recurrences in the longer term and a lower rate of adverse events when TNOM fails. To provide insight into the optimal care strategy for patients with aSBO, this study sought to provide a cost analysis of EOM compared with TNOM using real‐world population‐based data.

## Methods

This cost‐effectiveness study was based on population‐level data collected for patients admitted to hospital for aSBO in Ontario, Canada, in 2005–2014. The universally accessible, single‐payer healthcare system in Ontario permits population‐based studies with comprehensive longitudinal follow‐up and minimal selection bias. Data for this study were obtained using linked health administrative databases made available through the Institute for Clinical Evaluative Sciences (IC/ES). Costs were considered from the perspective of the healthcare payer, and a time horizon of 5 years was used. To account for the diminishing value of costs and benefits over time, discounting was applied to costs and benefits. Discounting reflects individual preferences for benefits to be experienced in the present rather than the future, and for costs to be experienced in the future rather than the present^13^.

### Cohort identification and data sources

All patients aged 18–80 years who were admitted to hospital in Ontario for their first episode of aSBO between 1 April 2005 and 31 March 2014 were identified using a validated algorithm to capture the index admission for aSBO using health administrative databases, as described previously^14^. All patients with an active cancer diagnosis were identified using administrative diagnostic codes and excluded from analyses.

Data were obtained from the following sources: Discharge Abstract Database (diagnostic and resource utilization data); Registered Persons Database (demographic data for all residents of Ontario); Ontario Health Insurance Plan (OHIP) Claims Database (physician‐submitted billing codes); Office of the Registrar General Database (cause of death data); and IC/ES‐derived cohorts for specific co‐morbidities (congestive heart failure, hypertension, myocardial infarction, chronic obstructive pulmonary disease and diabetes mellitus). Data from these sources have been validated previously for a wide range of surgical and non‐surgical diseases^15^.

The Discharge Abstract Database captures all associated and co‐morbid conditions for patients admitted to hospital. Any patient with an associated or co‐morbid condition that included any abdominal malignancy was excluded from the study to minimize the risk of misclassification of malignant bowel obstruction as adhesive bowel obstruction.

### Early operative management *versus* trial of non‐operative management

Patients managed by EOM were compared with those managed by TNOM. EOM was defined as operative intervention on either the calendar day of admission or the calendar day after admission. TNOM was defined as the management approach when the patient did not undergo operative intervention on the day of admission or the day after admission. Thus, patients in the TNOM group were either successfully managed without surgery or had a failure of non‐operative management and underwent delayed surgery (operative intervention more than 1 calendar day after admission).

Hospitals in Ontario may have individual protocols or preferences with regard to the management of aSBO, but there are no standardized protocols that are implemented province‐wide. Considerable variability exists between hospitals across the province in the management of aSBO, particularly with respect to the proportion of patients who undergo surgery and the timing of surgery^16^.

### Outcomes

#### Incremental cost‐effectiveness ratio at 5 years

The effectiveness of an intervention was measured in terms of quality‐adjusted life expectancy (QALE). QALE is a measure of survival that is adjusted for quality of life and the ongoing burden of disease by accounting for time spent in less than optimal health states. Each quality‐adjusted life‐year (QALY) is anchored on a scale where the value of 1 represents 1 year lived in perfect health and 0 represents death^17^. The primary outcome was the incremental cost‐effectiveness ratio (ICER) of EOM compared with TNOM after 5 years of follow‐up. The ICER of one intervention over another is a measure of the cost‐per‐unit of effectiveness with that intervention. ICER was reported in terms of dollars per QALY gained by EOM ($/QALY (€/QALY)). All costs were adjusted to 2014 Canadian dollars (2014 euros).

#### Clinical outcomes

Estimation of the ICER required calculation of QALYs and costs. As differences in QALYs and costs were driven by clinical events, the rates of recurrences of aSBO and associated adverse events in each treatment group were reported. Recurrences were defined as admissions for aSBO that began at least 30 days after discharge from the previous admission for aSBO. Operative intervention and its timing, and the incidence of serious complications and/or bowel injury or resection, were identified using claims data and discharge abstracts, as described previously^14^. The causes of postoperative mortality were explored, and the overall survival of patients with aSBO was estimated.

#### Utilities

A utility is a measure of the value placed on a particular health state. QALYs are estimated by adjusting survival by the duration of time spent in suboptimal health states and the utility of each health state^17^. Utility scores associated with aSBO have been reported previously^18^, with non‐operative admissions being associated with a utility of 0·65 and operative admissions with a utility of 0·55. aSBO is an episodic condition, characterized by long intervals of normal quality of life, with relatively brief episodes of disutility. As such, the utility associated with non‐operative admissions was attributed for 7 days and the utility associated with operative admissions was attributed for 30 days, as has been done in previous studies of aSBO^18^.

No published studies specifically report the utilities associated with aSBO‐related complications. Acute Crohn's disease and ulcerative colitis have commonly been used as surrogate diseases in the estimation of aSBO utilities^19^, ^20^. Utilities associated with complications of acute ulcerative colitis have been reported using the time‐trade‐off method^21^. The weighted means (by sample size) of the utilities reported in these three studies^19^, ^20^, ^21^ were calculated to establish a value for the present study. For this study, the utility associated with complications was attributed for 30 days. Serious complications were defined based on the definitions used by the US National Surgical Quality Improvement Program (NSQIP)^22^. In instances of multiple concurrent utilities, the lowest utility score was used.

### Costs

Inpatient costs were evaluated from the perspective of the healthcare payer and calculated at the individual patient level using the resource intensity weight (RIW) method. Each hospitalization in Ontario is attributed a RIW value, which is a measure of the resource use (and costs). RIW is calculated based on patient age, co‐morbidity burden, primary diagnosis for the admission, interventions received, and the length of hospital stay^23^. Costing for this study utilized the provincial‐average unit costs rather than hospital‐specific costs, which may vary between treatment centres. The resulting costs represent the total inpatient‐related costs associated with each admission for aSBO at the patient level and include direct medical as well as overhead hospital costs.

### Statistical analysis

#### Propensity score matching

Patient‐level co‐variables were used in a multivariable logistic regression to generate a propensity score for each patient, reflecting the likelihood of being managed by EOM. Patients managed by EOM were matched 1 : 1 by propensity score to patients managed by TNOM, using a ‘greedy’ algorithm and a caliper width of 0·2 of the standard deviation of the logit of the propensity score^24^. Balance diagnostics were performed to ensure that the resulting cohort was well matched based on standardized differences. A standardized difference greater than 10 per cent was considered significant^25^. Potential confounders to the relationship between management strategy (TNOM *versus* EOM) and outcomes were identified and included in propensity score matching. Patient characteristics included age, sex, co‐morbidity burden, socioeconomic status (income quintile) and rurality (Rurality Index of Ontario, a measure of population density as well as the distance to the nearest referral centres)^26^.

#### Clinical outcomes contributing to cost‐effectiveness

Patients were followed over a 5‐year time horizon to determine the number of recurrences and aSBO‐related events, as well as survival and accumulated inpatient costs. aSBO‐related events included operations for aSBO, bowel resections or injuries, and serious complications. The overall survival of patients with aSBO was estimated using the Kaplan–Meier method, and survival curves were compared between treatment groups using the log rank test. The data available captured all admissions from 2005 to 2014. To avoid right censoring and ensure at least 5 years of follow‐up for all patients, the primary analysis included all patients admitted for their index episode from 2005 to 2010.

#### Incremental cost‐effectiveness ratio at the 5‐year time horizon

The mean 5‐year discounted ICER ($/QALY (€/QALY)) for EOM compared with TNOM was calculated as the primary outcome. This calculation was performed by dividing the difference in mean costs between EOM and TNOM by the difference in mean QALY. Costs and utilities were discounted at a rate of 1·5 per cent per year, as per guidelines published by the UK National Institute for Health and Care Excellence^13^, ^27^.

#### Sensitivity analyses

The change in ICER over time was also calculated, to quantify how the cost‐effectiveness of EOM changes with longer follow‐up. The ICER at different time horizons (1 year, 2 years, up to 7 years) was plotted. For each time horizon used, all patients for whom necessary follow‐up data were available were included. The analysis was also repeated after excluding patients who were taken directly to an operating room or ICU from the emergency department. These patients likely represented those for whom a trial of non‐operative management may not have been possible.

All analyses were performed using SAS® Enterprise Guide version 7.1 (SAS Institute, Cary, North Carolina, USA). Results were considered statistically significant when *P* < 0·050.

## Results

Over the study period, 25 150 patients were admitted to 122 hospitals in Ontario for their first episode of aSBO. Some 23·7 per cent of patients were admitted to hospitals classified as large teaching hospitals (university‐affiliated hospitals with more than 100 beds – group A hospitals under the Ontario Public Hospitals Act), whereas 53·2 per cent were admitted to large non‐teaching hospitals (those with more than 100 beds – group B hospitals), and 23·7 per cent were admitted to non‐teaching hospitals with fewer than 100 beds (group C hospitals). Nine hospitals were trauma centres, and all were public hospitals.

The mean(s.d.) patient age was 61·2(13·7) years, and 51·1 per cent of patients were women. Some 3174 patients (12·6 per cent) were managed by EOM, and the others by TNOM. Patients managed by EOM were slightly younger (mean age 58·9 years *versus* 61·5 years for the TNOM group), had fewer co‐morbidities, lived in more urban areas, and were treated in larger hospitals. Overall, TNOM was unsuccessful in 11·7 per cent of patients, who underwent a delayed operation at a median of 3 (i.q.r. 2–5) days after admission.

**Fig. 1:**
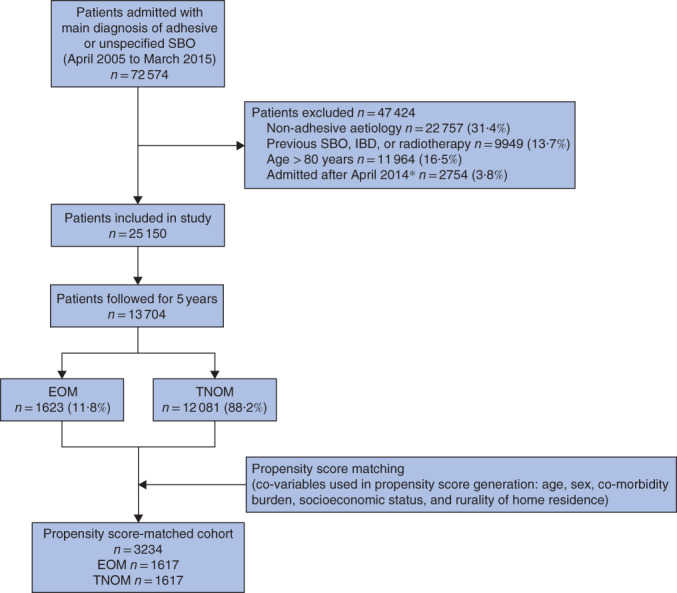
Flow diagram of patient eligibility and study design*Patients admitted after April 2014 were excluded to allow follow‐up of at least 1 year for all patients. SBO, small bowel obstruction; IBD, inflammatory bowel disease; EOM, early operative management; TNOM, trial of non‐operative management.

A total of 13 704 patients had 5 years of follow‐up, among whom 1623 (11·8 per cent) were managed by EOM. After propensity score matching, a matched cohort of 3234 patients (1617 in each group) was used for subsequent analyses (*Fig*. *1*). Balance diagnostics suggested that the groups were well matched (*Table* *1*). Some 10·1 per cent of patients who were initially managed by TNOM in the matched cohort had failure of non‐operative management and underwent a delayed operation.

**Table 1:** Baseline characteristics of the matched cohort

	Overall (*n* = 3234)	TNOM (*n* = 1617)	EOM (*n* = 1617)	Standardized difference
Age (years)*	59·0(14·7)	59·2(14·3)	58·9(14·4)	0·02
**Women**	1782 (55·1)	897 (55·5)	885 (54·7)	0·02
**Co‐morbidity**				
MI within last 2 years	19 (0·6)	6 (0·4)	13 (0·8)	0·05
Hypertension	1556 (48·1)	783 (48·4)	773 (47·8)	0·01
CHF	213 (6·6)	100 (6·2)	113 (7·0)	0·03
Diabetes mellitus	621 (19·2)	304 (18·8)	317 (19·6)	0·02
COPD	647 (20·0)	313 (19·4)	334 (20·6)	0·05
**Income quintile**				
1st (lowest)	655 (20·3)	330 (20·4)	325 (20·1)	< 0·01
2nd	630 (19·5)	307 (19·0)	323 (20·0)	0·03
3rd	647 (20·0)	328 (20·3)	319 (19·7)	0·02
4th	658 (20·3)	327 (20·2)	331 (20·5)	< 0·01
5th	644 (19·9)	325 (20·1)	319 (19·7)	0·01
**Rural residence**	137 (4·2)	63 (3·9)	74 (4·6)	0·03
**Teaching hospital**	876 (27·1)	433 (26·8)	443 (27·4)	0·01
**Large hospital**	1581 (48·9)	774 (47·9)	807 (49·9)	0·04

Values in parentheses are percentages unless indicated otherwise; *values are mean(s.d.). TNOM, trial of non‐operative management; EOM, early operative management; MI, myocardial infarction; CHF, congestive heart failure; COPD, chronic obstructive pulmonary disease.

**Fig. 2:**
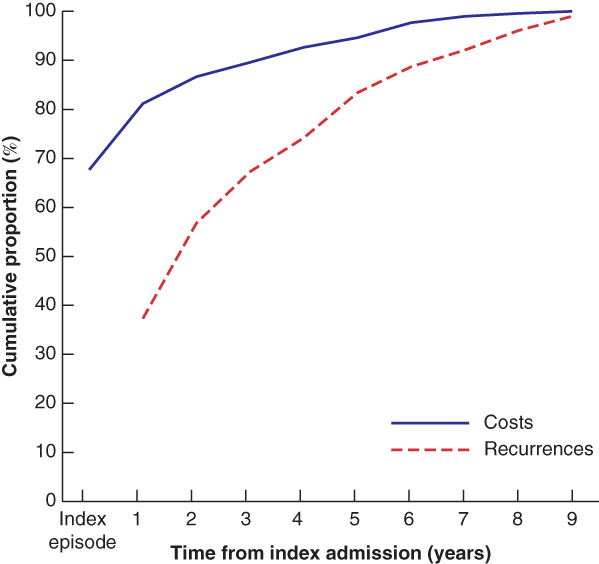
Accumulation of costs and recurrences over time

The maximum duration of follow‐up was 9 years. The accumulation of aSBO recurrences and associated costs over time were plotted using the subgroup of patients with 9 years of follow‐up as a reference. After 5 years' follow‐up, 83·4 per cent of recurrences and 94·6 per cent of aSBO‐related costs had been incurred (*Fig*. *2*).

For reference, and to allow comparison with other healthcare systems, the mean costs of individual admissions for aSBO are shown in *Table* *S1* (supporting information).

### Clinical outcomes contributing to the cost analysis

#### Recurrences and utilities

The overall cumulative incidence of aSBO recurrence within 5 years was significantly greater in patients managed by TNOM than in those managed by EOM (20·9 *versus* 13·2 per cent respectively; *P* < 0·001). Patients who had one recurrence were more likely to experience additional, subsequent recurrences. As a cohort, patients in the TNOM group thus experienced 82 per cent more recurrences than those in the EOM group (593 *versus* 325 respectively; mean 0·37 *versus* 0·20 recurrences per patient, *P* < 0·001). As a consequence, patients managed by TNOM also experienced more aSBO‐related events over the course of follow‐up.

aSBO‐related events included bowel resection/injury and serious complications. Patients in the TNOM group experienced fewer serious complications than those in the EOM group (6·1 *versus* 10·0 per cent respectively; *P* < 0·001). However, when comparing patients who underwent surgery (patients in the EOM group *versus* those in the TNOM group who failed non‐operative management and had a delayed operation), the risk of serious complications was similar (10·0 *versus* 12·9 per cent respectively; *P* = 0·243). Patients in both groups who underwent surgery had a similar risk of bowel injury and/or requiring a bowel resection (43·5 per cent in the EOM group *versus* 45·4 per cent in the TNOM group; *P* = 0·647).

After attributing the utilities associated with aSBO‐related events that occurred at the index admission and during follow‐up, the resulting quality‐adjusted life expectancy in the two groups demonstrated an overall net effectiveness associated with EOM: 4·51 QALY *versus* 4·30 QALY for TNOM, of a maximum possible 5 QALY; *P* < 0·001).

#### Survival

**Fig. 3:**
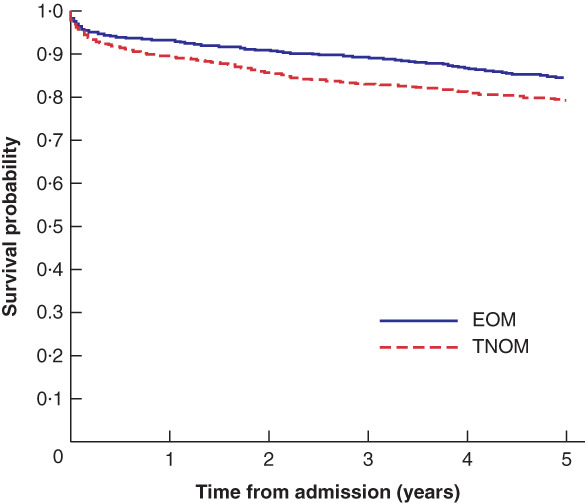
Kaplan–Meier survival curves for early operative management and trial of non‐operative management groupsEOM, early operative management; TNOM, trial of non‐operative management. *P* < 0·001 (log rank test).

The primary driver of the difference in QALY was a survival benefit associated with EOM. There was a significant difference in mean survival between the two groups, with patients managed by EOM experiencing significantly greater mean survival at 5 years of follow‐up than those in the TNOM group (4·6 *versus* 4·3 years respectively; *P* < 0·001). Kaplan–Meier curves comparing survival between the two study groups are presented in *Fig*. *3*. Log rank testing of the Kaplan–Meier survival function demonstrated significantly longer survival in the EOM group (*P* < 0·001), with an adjusted hazard ratio for death of 0·72 (95 per cent c.i. 0·61 to 0·85) associated with EOM.

The overall risk of dying within 90 days of admission for aSBO was 7·9 per cent. The 90‐day mortality rate was significantly greater in the TNOM group than in the EOM group (8·9 *versus* 6·8 per cent respectively; *P* = 0·026).

Using available cause of death data for the entire (unmatched) cohort, among patients who died within 90 days of admission the underlying cause of death was reported as SBO in 29·1 per cent of deaths. The non‐SBO causes of death for the remainder of 90‐day mortalities closely resembled the age‐related causes of death for the general population.

### Costs

After 5 years of follow‐up, patients managed by EOM had incurred greater mean costs ($17 951 *versus* $11 594 (€12 288 *versus* €7936) for patients managed by TNOM; *P* < 0·001). The majority of costs in both groups were accrued during the index episode. However, for patients managed by TNOM, 30·0 per cent of total costs were incurred during recurrences, compared with 10·7 per cent for patients managed by EOM.

### Incremental cost‐effectiveness ratio at 5 years

After 5 years of follow‐up, EOM was associated with a net utility benefit of 0·21 QALY, at an overall cost difference of $6352 (€4348). The associated ICER for EOM compared with TNOM was $29 881 (€20 454) per QALY. The unadjusted cost per absolute life‐year gained was similar to the cost per QALY gained ($26 737 (€18 302) per year).

#### Sensitivity analyses

**Fig. 4:**
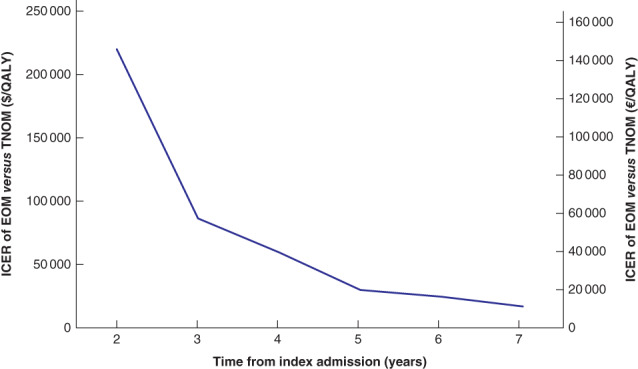
Incremental cost‐effectiveness ratio for early operative management *versus* trial of non‐operative management over timeICER, incremental cost‐effectiveness ratio; EOM, early operative management; TNOM, trial of non‐operative management; QALY, quality‐adjusted life‐year.

**Fig. 5:**
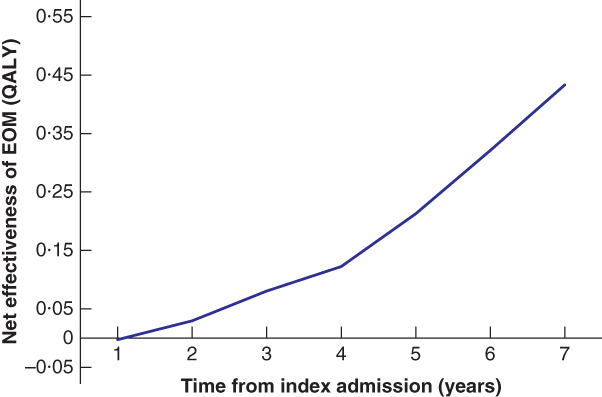
Net effectiveness of early operative management over timeEOM, early operative management; QALY, quality‐adjusted life‐year.

With each additional year of follow‐up, the ICER associated with EOM compared with TNOM decreased, suggesting improved cost‐effectiveness ($219 847 (€150 489) per QALY with 2 years of follow‐up to $16 887 (€11 559) per QALY with 7 years) (*Fig*. *4*). The differences in QALYs increased, as longer follow‐up allowed for more recurrences and greater differences in survival (difference of 0·03 QALY with 2 years of follow‐up to 0·43 QALY with 7 years) (*Fig*. *5*).

After excluding patients who were taken directly from the emergency department to either an operating room or an ICU, the ICER after 5 years was similar to that of the cohort used in the primary analysis ($31 774 (€21 750) *versus* $29 881 (€20 454) per QALY respectively).

## Discussion

This retrospective cost–utility analysis used administrative data to compare two competing strategies for the management of aSBO: EOM and TNOM. Using population‐level administrative data, the analyses suggest that, when considering the long‐term outcomes of aSBO, EOM may be a cost‐effective approach to care within 5 years of the index episode. Although EOM is more expensive, it is associated with fewer recurrences and longer survival. The cost–utility of EOM appears to be mediated through prevention of recurrences and recurrence‐associated adverse events.

The findings of the present study suggest a few considerations for the management of aSBO. The approach endorsed by current guidelines^4^ focuses on an individual admission and may not consider the long‐term consequences of different management approaches. However, the decision‐making for aSBO may benefit from considering the natural history of the disease, rather than only the acute admission.

Current guidelines may underestimate the risk of recurrence with TNOM, and the morbidity and mortality associated with each admission for aSBO. In this study, there was a 90‐day mortality rate of 7·9 per cent. Several recent population‐based studies^9^, ^28^, ^29^, ^30^ have found similar short‐term mortality rates associated with aSBO, ranging from 6·2 to 7·2 per cent.

The evaluation of whether or not an intervention is cost‐effective relies on defining health benefits in monetary terms within a distinct socioeconomic context and assuming underlying judgement about value^31^. Although a wide range of willingness‐to‐pay thresholds have been used, the ICER for EOM of $29 881 (€20 454) is well below all recently published thresholds for uptake of an intervention^31^. A recent study^32^ that used Medicare reimbursements reported costs associated with non‐operative and operative admissions for aSBO of $6174 (€4226) and $21 199 (€14 511) respectively after conversion to 2014 Canadian dollars. The corresponding values for the cohort used in this study were $6717 (€4598) and $19 896 (€13 619).

The results of this study must be interpreted in the context of the data that were available. The study relied on administrative health data, rather than clinical data. Many of the administrative data used were not captured for the purpose of clinical research, particularly not for clinical research of this specific disease process. Although each database used for this study has been validated against chart review, the use of administrative data is inherently associated with misclassification bias. Moreover, the non‐clinical nature of the data prohibited the identification of many important variables, including preoperative clinical details of the SBO episode, the extent of SBO, surgical data, and granular postoperative clinical data. Future studies examining long‐term outcomes of aSBO that are able to use clinical data will be of considerable value to this body of work.

This study benefited from the large, unselected data set, which allowed for longitudinal follow‐up with minimal loss. Access to patient‐level costing data allowed for the estimation of total costs over follow‐up for each patient, accounting for all downstream aSBO‐related events. A matching approach was used to mitigate potential confounding by indication, and results were robust to sensitivity analysis. However, as with any analysis that relies on retrospective data, residual unmeasured confounding and the risk of misclassification bias invariably exist. In addition, data regarding the previous surgical history of patients in the cohort were not available. The type of surgery and time elapsed from previous surgery may vary between study groups.

Another limitation of this study is the paucity of literature regarding the utility scores associated with aSBO. Ultimately, the uncertainty surrounding utility parameters does not have a significant impact on the findings. As aSBO is characterized by relatively brief episodes and long periods of normal quality of life, the accumulated disutility associated with aSBO was quite small, regardless of management approach. The primary reason for the net effectiveness of EOM was the survival benefit, and this was unaffected by the uncertainty surrounding utility estimates. A recently published study^10^ explored the survival benefit associated with operative management of aSBO using a combination of propensity score methods and an instrumental variable analysis to account for unmeasured confounding.

Finally, granular data of non‐operative interventions, such as the use or duration of nasogastric suction, inpatient medication use, and use of cantor tubes, was not available. Data regarding the use of water‐soluble contrast studies were also unavailable. Although water‐soluble contrast studies may reduce the need for surgery^33^, the findings suggest that successful non‐operative management of patients with aSBO may be detrimental in the long term.

This retrospective study of administrative data indicated that early operative management may result in fewer recurrences and represent a cost‐effective approach to patients with aSBO.

## Supporting information


**Table S1** Inpatient costs associated with admissions for adhesive small bowel obstruction
