# Supplementary material for: Early operative management in patients with adhesive small bowel obstruction: population‐based cost analysis
Source: BJS Open. 2020 Jun 30;4(5):914–23. doi: 10.1002/bjs5.50311 (PMC7528511; doi:10.1002/bjs5.50311)
Supplement: Supplementary file 1 — Table S1 Inpatient costs associated with admissions for adhesive small bowel obstruction [file BJS5-4-914-s001.docx]

**BJS5_50311**

**Early operative management in patients with adhesive small bowel obstruction: population-based cost analysis**

**R. Behman, A. B. Nathens, P. Pechlivanoglou, P. Karanicolas, J. Jung and N. Look Hong**

**Table S1** Inpatient costs associated with admissions for adhesive small bowel obstruction

|  | **Unmatched Cohort** | | **Matched Cohort** | |
| --- | --- | --- | --- | --- |
|  | **Mean Cost ($)** | **Mean Cost (€)** | **Mean Cost ($)** | **Mean Cost (€)** |
| **All Index Non-operative Episodes** | 6,717 | 4,598 | 6,729 | 4,606 |
| No complication | 5,747 | 3,934 | 5,585 | 3,823 |
| With Complication | 22,468 | 15,380 | 27,125 | 18,568 |
|  |  |  |  |  |
| **All Index Operative Episodes** | 19,896 | 13,619 | 16,428 | 11,245 |
| No complication, no bowel resection | 12,626 | 8,643 | 10,360 | 7,092 |
| With Complication, no bowel resection | 45,461 | 31,119 | 41,142 | 28,162 |
| No complication, with bowel resection | 18,907 | 12,942 | 16,203 | 11,091 |
| With Complication and bowel resection | 62,272 | 42,626 | 52,296 | 35,798 |
